# The impact and potential mechanisms of long noncoding RNA ENST00000521141.1 on human white adipocyte differentiation

**DOI:** 10.1530/JOE-25-0360

**Published:** 2026-04-21

**Authors:** Qiong Wu, Lianghui You, Shibo Lin, Tingting Yu, Rui Yin, Xinrong Ye, Lijian Xu, Hui Liang, Lingxia Pang

**Affiliations:** ^1^ Nanjing Women and Children’s Healthcare Institute, Women’s Hospital of Nanjing Medical University, Nanjing Women and Children’s Healthcare Hospital, Nanjing, Jiangsu, China; ^2^ Department of General Surgery, Nanjing Tianyinshan Hospital & The Affiliated Hospital of China Pharmaceutical University, The First Affiliated Hospital of China Pharmaceutical University, Nanjing, Jiangsu, China; ^3^ Department of General Surgery, Division of Bariatric & Metabolic Surgery, First Affiliated Hospital with Nanjing Medical University, Nanjing, Jiangsu, China; ^4^ Department of General Surgery, The Second Affiliated Hospital of Nanjing Medical University, Nanjing, China; ^5^ Children’s Healthcare Department, Women’s Hospital of Nanjing Medical University, Nanjing Women and Children’s Healthcare Hospital, Nanjing, Jiangsu, China; ^6^ Nanjing Medical Key Laboratory of Developmental Behavioral Pediatrics, Women’s Hospital of Nanjing Medical University, Nanjing Women and Children’s Healthcare Hospital, Nanjing, Jiangsu, China

**Keywords:** lncRNA, lncRNA521141, human preadipocytes, adipogenic differentiation, PDGFRA

## Abstract

Accumulation of visceral white adipose tissue leads to central obesity and is associated with insulin resistance and increased risk of metabolic disease. lncRNAs have been reported to regulate the growth and development of adipocytes, providing new clues for the prevention and treatment of obesity. In the present study, we identified numerous dysregulated lncRNAs by microarray analysis between human differentiated adipocytes and preadipocytes. In addition, we focused on the lncRNA ENST00000521141.1 (abbreviated as lncRNA521141) confirmed by qRT-PCR to be enriched in differentiated adipocytes. Lentivirus-mediated knockdown of lncRNA521141 significantly suppressed adipogenesis as evidenced by reduced lipid accumulation, triglyceride content, and key adipogenic markers expression, while lentivirus-mediated lncRNA521141 overexpression showed no significant effect. Given that lncRNA521141 did not alter the expression of the neighboring gene PEBP4, fluorescence *in situ* hybridization was employed to elucidate the cellular localization of lncRNA521141, revealing its predominant distribution in the nucleus. RNA-sequencing and western blot analysis further revealed the potential involvement of the PI3K-Akt and thyroid hormone synthesis pathways in lncRNA521141-mediated adipogenic inhibition. Among the differentially expressed genes, PDGFRA expression was significantly upregulated following lncRNA521141 silencing and was closely associated with adipocyte differentiation. Rescue experiments confirmed that knockdown of PDGFRA expression after lncRNA521141 silencing alleviated the inhibitory effects of the reduced lncRNA521141 expression on adipocyte differentiation. In summary, the present study identified lncRNA521141 as a key regulator of adipogenic differentiation in human preadipocytes and elucidated its potential regulatory mechanism, thereby providing new insights and potential intervention targets for treating obesity and its related diseases.

## Introduction

With the development of the economy and lifestyle changes, the global obesity population has significantly increased. Obesity and related metabolic diseases have become a serious social burden, endangering the physical and mental health of individuals. As a chronic recurrent disease, obesity is an important risk factor for hypertension, coronary artery disease and stroke, type 2 diabetes mellitus, and other metabolic and malignant diseases (1). According to the World Health Organization, 2.5 billion adults were overweight in 2022, including 890 million with obesity, highlighting an urgent need for effective prevention and control strategies.

The main manifestations of obesity are excessive accumulation of white adipose tissue (WAT), with increased local content and abnormal distribution (2). As a major energy storage depot and important endocrine organ, WAT plays a pivotal role in systemic metabolic regulation (3). Preferential accumulation of visceral WAT is associated with an increased risk of insulin resistance, whereas subcutaneous WAT expansion is protective (4). Adipogenesis of visceral WAT, involving precursor cell proliferation, adipogenic lineage commitment, and terminal differentiation (5), is critical for whole-body energy homeostasis, and its dysregulation leads to lipid over-accumulation and obesity (6). Thus, clarifying the molecular mechanisms of adipogenesis is essential for developing anti-obesity therapies.

Numerous transcription factors and signaling pathways are involved in adipocyte differentiation (7). Among these, CAAT/enhancer-binding protein α (C/EBPα) and peroxisome proliferator activated receptor γ (PPARγ) are the core transcriptional regulators, which synergistically induce the expression of adipogenic-related genes (8). Key signaling pathways of adipocyte differentiation include the Wnt, transforming growth factor beta (TGF-β) superfamily, and cyclic adenosine monophosphate (cAMP) pathways (8, 9, 10). These findings lay a foundation for understanding adipocyte differentiation and obesity pathogenesis.

Long noncoding RNAs (lncRNAs) are non-protein-coding RNA molecules widely present in eukaryotes, and they are critical for various biological processes (11, 12, 13). Emerging evidence shows that lncRNAs are extensively involved in WAT metabolism and adipocyte differentiation across species (14). For example, the lncRNA Gm15290 promotes adipogenesis in mice (15), while bovine lncRNAs, such as lncCCPG1, facilitate adipocyte differentiation (16). In addition, adipocyte differentiation-associated long noncoding RNA (ADNCR) exerts anti-adipogenic effects (17). Our previous study also identified many lncRNAs and validated the effects of the lncRNA Gm13133 on the differentiation of mouse white adipocytes (18). Because lncRNAs exhibit poor evolutionary conservation (19), the annotation and functional exploration of human adipocyte-derived lncRNAs are valuable for elucidating obesity mechanisms in humans and identifying potential therapeutic targets.

The present study identified differentially expressed lncRNAs between human adipocytes (Ad group) and preadipocytes (preAd group) via microarray analysis. After confirmation of lncRNA521141 differential expression, the role and regulatory mechanisms of this lncRNA in adipogenesis were explored. Functional assessments revealed that knockdown of lncRNA521141 significantly inhibited adipogenic differentiation of human white adipocytes, whereas its overexpression had no obvious effect. Further study indicated that lncRNA521141 may modulate the adipogenic program by targeting platelet-derived growth factor receptor alpha (PDGFRA). Collectively, the present findings identified lncRNA521141 as an essential regulator of human preadipocyte differentiation, providing a potential therapeutic target for obesity and related metabolic disorders.

## Materials and methods

### Cell culture and differentiation

Human preadipocytes (before passage 5) derived from visceral WAT were thawed and maintained in preadipocyte growth medium (PAM) supplemented with 5% fetal bovine serum, 1% growth supplement, and 1% penicillin/streptomycin solution (ScienCell Research Laboratories, USA). Upon reaching confluence, the cells were cultured for an additional 1–2 days. Subsequently, the preadipocytes were induced to differentiate using Dulbecco’s modified Eagle’s medium/nutrient mixture F-12 (DMEM/F-12; Gibco, USA) containing 0.5 mM 3-isobutyl-l-methylxanthine, 1 μM dexamethasone, 860 nM insulin, 1 μM rosiglitazone, and 1% penicillin/streptomycin solution (all from Sigma-Aldrich, USA) for 4 days. The medium was then replaced with the maintenance medium consisting of DMEM/F-12 (Gibco), 100 nM insulin (Sigma-Aldrich), and 1% penicillin/streptomycin solution until the adipocytes were fully mature, as indicated by the accumulated lipid droplets. These adipocytes were collected on days 0, 1, 4, and 10 of the differentiation process for further analysis.

### Construction and generation of lentivirus vectors and their infection with human preadipocytes

The lentiviral vectors for overexpression and silencing of lncRNA521141 were obtained from GenePharma Co. (Shanghai, China). When the cell confluence reached 30–40% (at a density of approximately 3 × 10^5^ cells), the culture medium was supplemented with either the lncRNA521141-overexpressing (lnc-OV) or lncRNA521141-silencing (lnc-KD) lentivirus, along with their respective negative control lentivirus (NC) and polybrene (5 μg/mL), all at a viral titer of approximately 0.6 × 10^6^ UT/mL for lnc-OV and 2.5 × 10^6^ UT/mL for lnc-KD. After 16–18 h of transduction, the cells were supplied with fresh growth medium and cultured to induce adipogenesis, as described in the section titled Cell culture and differentiation.

### RNA extraction, reverse transcription, and quantitative real time-polymerase chain reaction (qRT-PCR)

Total RNA was extracted from adipocytes using TRIzol reagent (Invitrogen, USA). Subsequently, the RNeasy Mini Kit (Tiangen Biotech, China) was employed to purify the RNA according to the manufacturer’s instructions. For each sample, 2,000 ng of RNA were reverse-transcribed into cDNA using the HiScript II Q RT SuperMix for qPCR (plus gDNA wiper) (Vazyme, China). qRT-PCR was performed on the QuantStudio™ 7 Flex Real-Time PCR System (Applied Biosystems, USA) using the SYBR Green method (Life Technologies Corp, USA), following the manufacturer’s protocols. The relative expression levels of genes were normalized to the internal reference peptidylprolyl isomerase A (PPIA) using the 2^-△△Ct^ method. All primers used in the study are listed in Supplementary Table S1 (see section on Supplementary materials given at the end of the article).

### Protein extraction and western blot

Total protein was extracted from adipocytes using RIPA lysis buffer (Beyotime Biotechnology, China) supplemented with 2% protease inhibitor cocktail (Roche, Switzerland). The protein concentration was determined using a BCA assay kit (Thermo Fisher Scientific, USA) according to the manufacturer’s protocol. For immunoblotting, protein samples were separated by sodium dodecyl sulfate-polyacrylamide gel electrophoresis (SDS-PAGE) using 8, 10, or 12% gels based on the molecular weight of target proteins. The separated proteins were then transferred to a PVDF membrane (Millipore Corp., USA), and the membranes were blocked and then probed with specific primary antibodies as follows: anti-C/EBPα (Abcam, UK; Cat. No. ab40764; diluted 1:1,000), anti-C/EBPβ (Abcam; Cat. No. ab32358; diluted 1:1,000), anti-PPARγ (Abcam; Cat. No. ab178860; diluted 1:1,000), anti-FABP4 (Abcam; Cat. No. ab40764; diluted 1:1,000), anti-PEBP4 (ImmunoWay, USA; Cat. No. YT6502; diluted 1:1,000), anti-HGF (Proteintech, China; Cat. No. 26881-1-AP; diluted 1:1,000), anti-PDGFRA (CST; Cat. No. 3174T; diluted 1:1,000), anti-GPX3 (Abcam; Cat. No. ab275965; diluted 1:1,000), anti-TSHR (Proteintech; Cat. No. 14450-1-AP; diluted 1:1,000), and anti-GAPDH (Biosharp; Cat. No. BL005B; diluted 1:1,000). The secondary antibodies used were goat anti-rabbit IgG HRP (Biosharp; Cat. No. BL003A; diluted 1:1,000). The gray values were scanned and calculated with ImageJ software (National Institutes of Health, USA).

### Oil Red O staining

Lipid accumulation was assessed by Oil Red O staining on day 10 of the differentiation program. Adipocytes were washed twice with PBS and then fixed for 30 min with 4% paraformaldehyde (Biosharp, China). After fixation, the cells were washed twice with PBS, stained with 0.3% (m/v) Oil Red O (Sigma-Aldrich) dissolved in isopropanol (Damao Chemical Reagent Factory, China) for 30 min at 37°C. The lipid droplets were visualized using the Imager A2 fluorescence microscope (Carl Zeiss, Germany), and representative images are shown.

### Triglyceride detection

Triglyceride accumulation was assessed on day 10 of the adipocyte differentiation program using a chemiluminescence method with the triglyceride content assay kit (Cat. No. E1013, Applygen, China) according to the manufacturer’s instructions.

### Fluorescence *in situ* hybridization (FISH)

The differentiated adipocytes were fixed with 4% paraformaldehyde and then permeabilized using PBS containing 0.5% Triton X-100 to enhance probe accessibility. After washing with PBS, the cells were subjected to pre-hybridization for 1 h at 37°C to reduce nonspecific binding. Hybridization was performed overnight with a hybridization solution containing Cy3-labeled lncRNA FISH Probe Mix and positive probes (U6) (Ribobio, China). Following hybridization and rinsing, the cells were stained with DAPI to visualize the nuclei. Representative images were captured using the Leica STELLARIS STED confocal microscope (Leica Microsystems, Germany).

### RNA-sequencing and bioinformatics analysis

On day 10, RNA from differentiated adipocytes in the NC and lncRNA521141 knockdown (lnc-KD) groups was collected using TRIzol reagent. The transcriptome sequencing and subsequent bioinformatics analysis were performed by Novogene Co., Ltd (China). Briefly, the total amount and integrity of RNA were determined using the RNA Nano 6000 Assay Kit and Bioanalyzer 2100 system (Agilent Technologies, USA). Next, total RNA was used as an input material for library preparation. First, mRNA was purified from total RNA by using poly-T oligo-attached magnetic beads. After fragmentation, first-strand cDNA synthesis, and RNase H treatment for RNA degradation, second-strand cDNA synthesis was performed using DNA Polymerase I and dNTPs. Following adenylation of 3′ ends of DNA fragments and ligation of adapters, the library fragments were purified and obtained with the AMPure XP system (Beckman Coulter, USA). After the library was qualified, it was sequenced using the Illumina NovaSeq 6000. Subsequently, the raw sequence data (reads) were processed into high-quality clean data under quality control. The clean data were mapped to the reference genome using Hisat2 (v2.0.5) and quantified to FPKM by featureCounts (v1.5.0-p3). Differentially expressed genes (DEGs) were analyzed using the DESeq2 R package (1.20.0) and the following criteria: log2 |fold change| ≥ 1 and *P* < 0.05. The clusterProfiler R package (3.8.1) was used to test the statistical enrichment of differentially expressed genes in Kyoto Encyclopedia of Genes and Genomes (KEGG) pathways.

### Statistical analysis

The data analysis was performed using IBM SPSS statistics 20.0, and the results are expressed as mean ± standard error of the mean. Comparisons were made using an unpaired two-tailed *t*-test, and *P* < 0.05 was considered statistically significant.

## Results

### lncRNA521141 is identified by microarray screening and exhibits adipogenic-stage-specific expression

To uncover lncRNAs that are dynamically regulated during human adipogenesis, we compared the transcriptomes of undifferentiated preadipocytes (preAd) from the visceral depot with those of their fully differentiated adipocytes (Ad). lncRNA microarray profiling revealed 2,135 upregulated and 2,938 downregulated lncRNAs (fold change >2, *P* < 0.05; Supplementary Fig. S1A and S1B). After integrating the differentially expressed lncRNAs with the cognate mRNA signature, GO and KEGG analysis were performed. The top GO terms included lipid metabolism, lipid biosynthetic process, and cell differentiation, while the enriched pathways comprised PPAR, PI3K-Akt, Wnt, TGF-β, mitogen-activated protein kinase (MAPK), and adipogenic–lipogenic cascades (Supplementary Fig. S1C, D, F), implying a regulatory role for lncRNAs in human adipocyte differentiation. The 12 most abundant lncRNAs from the top 300 up- or downregulated lncRNAs (Supplementary Tables S2 and S3) were screened, and qRT-PCR analysis confirmed the microarray trends for all candidate lncRNAs (Supplementary Fig. S1G). Among them, ENST00000521141.1 (abbreviated as lncRNA521141) was markedly enriched in differentiated adipocytes. To our knowledge, the expression kinetics and functional relevance of lncRNA521141 in human adipogenesis and white adipose homeostasis remain unexplored.

**Figure 1:**
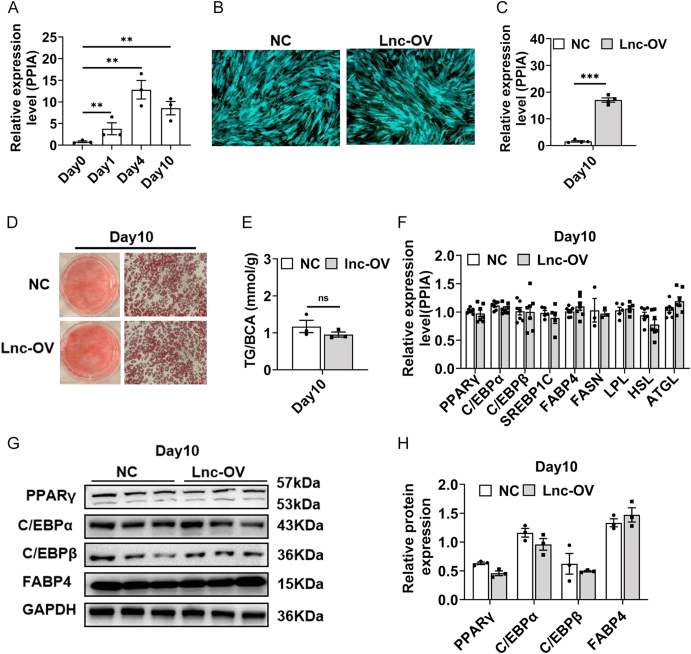
Overexpression of lncRNA521141 does not affect the adipogenic capacity of human preadipocytes. (A) lncRNA521141 expression levels in human preadipocytes were assessed by qPCR on days 0, 1, 4, and 10 of the differentiation program (three biologically independent samples per time point). (B) Fluorescence microscopy was used to evaluate the transfection efficiency of the lncRNA521141 overexpression lentivirus (lnc-OV) and the negative control lentivirus (NC). (C). The overexpression efficiency of lncRNA521141 was confirmed by qPCR (three independent experiments). (D) The effect of lncRNA521141 overexpression on the morphology and lipid droplet size of human adipocytes was examined by Oil Red O staining. (E) The impact of lncRNA521141 overexpression on triglyceride content was measured using a chemiluminescence assay (three biological replicates per group). (F) The adipogenic marker gene expression levels in the lnc-OV and NC groups on day 10 of the differentiation program were evaluated by qPCR (three to six independent experiments). PPIA mRNA levels served as an internal control for normalization. (G and H) The protein expression levels of human adipogenic markers, including PPARγ, C/EBPα, C/EBPβ, and FABP4, were evaluated by western blot analysis on day 10 of the differentiation program. Representative images are shown for each group (three biological replicates per group). Glyceraldehyde-3-phosphate dehydrogenase (GAPDH) protein levels were used as an internal control for normalization. The values are presented as the mean ± standard error of the mean (SEM). *P < 0.05, **P < 0.01, and ***P < 0.001. A full color version of this figure is available at https://doi.org/10.1530/JOE-25-0360.

The lncRNA521141 transcript, with a total length of 1,630 bp, is localized to the human 8p21.3 region. lncRNA521141 is classified as an antisense lncRNA, exhibiting a reverse complementary relationship with its neighboring gene, PEBP4 (Supplementary Fig. S2A). lncRNA521141 is relatively conserved in humans, rhesus monkeys, mice, dogs, and elephants, suggesting its potential functional importance (Supplementary Fig. S2B). Analyses using the Coding Potential Calculator and Coding Potential Assessment Tool revealed a low likelihood of protein-coding capacity for lncRNA521141 (Supplementary Fig. S2C). To verify the expression changes of lncRNA521141 during adipocyte differentiation, qRT-PCR was used to measure its expression level on days 0, 1, 4, and 10 of the differentiation process (Fig. 1A). Compared with those in undifferentiated preadipocytes (day 0), lncRNA521141 expression levels were significantly increased in differentiated adipocytes on days 1, 4, and 10, indicating that lncRNA521141 may participate in the regulation of adipogenic differentiation.

### Overexpression of lncRNA521141 does not influence human preadipocyte differentiation

To further elucidate the potential role of lncRNA521141 in adipocyte differentiation, lncRNA521141 was overexpressed in human preadipocytes using lentivirus transduction. Fluorescence microscopy confirmed high infection efficiency, and qRT-PCR analysis demonstrated a significant upregulation of lncRNA521141 in the overexpression group compared with the NC group (Fig. 1B and C). Notably, Oil Red O staining revealed no significant difference in lipid droplet size or morphology between lncRNA521141-overexpressing and control cells during differentiation on day 10 (Fig. 1D). Consistently, chemiluminescence assays revealed that lipid accumulation, as measured by triglyceride content, did not significantly differ between the two groups on day 10 (Fig. 1E). Moreover, the expression levels of key adipogenic genes were comparable between the control and the overexpression groups (Fig. 1F). Western blot analysis further confirmed that the protein expression of PPARγ, C/EBPα, C/EBPβ, and fatty acid-binding protein 4 (FABP4) were comparably expressed in both groups (Fig. 1G and H). Collectively, these findings demonstrated that overexpression of lncRNA521141 does not significantly impact adipogenic differentiation in human preadipocytes, suggesting that its functional role may be independent of this process.

### Knockdown of lncRNA521141 significantly impairs human preadipocyte differentiation

**Figure 2:**
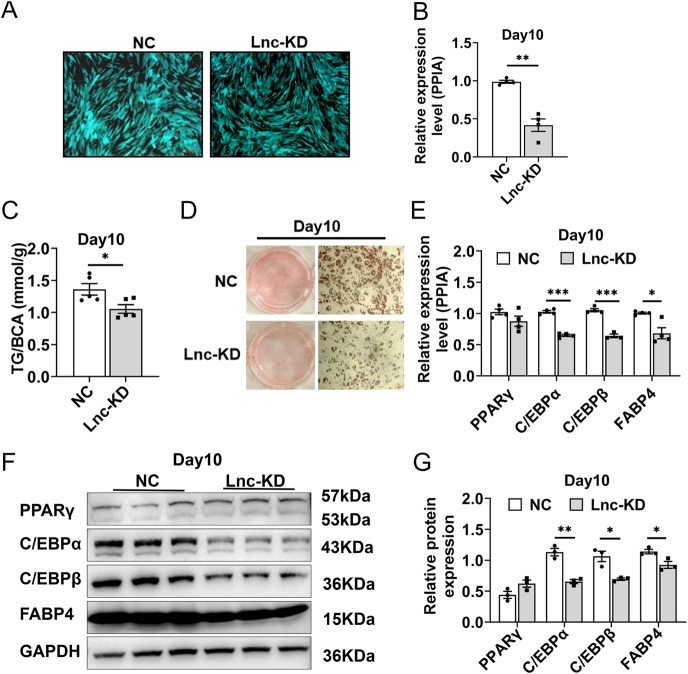
Knockdown of lncRNA521141 expression significantly inhibits the differentiation of human preadipocytes. (A) Fluorescence microscopy was used to assess the transfection efficiency in the lncRNA521141 knockdown (lnc-KD) and NC groups. (B) The efficiency of lncRNA521141 knockdown was confirmed by qPCR (four independent experiments). (C) The effect of lncRNA521141 knockdown on triglyceride content was measured using a chemiluminescence assay (five biological replicates per group). (D) Lipid accumulation was evaluated by Oil Red O staining in the lnc-KD and NC groups (three biological replicates per group). (E) The gene expression levels of adipogenic markers were compared between the lnc-KD and NC groups on day 10 of the differentiation program using qPCR (four independent experiments). PPIA mRNA levels were used as an internal control for normalization. (F and G) The protein expression levels of human adipogenic markers, including PPARγ, C/EBPα, C/EBPβ, and FABP4, were evaluated by western blot analysis on day 10 of the differentiation program. Representative western blot images are shown for each group (three biological replicates per group). GAPDH protein levels were used as an internal control for normalization. Values are presented as the mean ± SEM. *P < 0.05, **P < 0.01, and ***P < 0.001. A full color version of this figure is available at https://doi.org/10.1530/JOE-25-0360.

To investigate the role of lncRNA521141 in adipogenesis, siRNA-mediated gene silencing was employed in human preadipocytes. After screening multiple siRNA sequences, the most effective sequence (Si-3) was selected (Supplementary Fig. S3) and used to produce lentiviral vectors for subsequent functional analyses. Fluorescence microscopy confirmed high transfection efficiency, and qRT-PCR demonstrated significant lncRNA521141 knockdown (Fig. 2A and B). Chemiluminescence assays revealed a marked reduction in triglyceride accumulation in knockdown cells compared with the control cells (Fig. 2C). Similarly, Oil Red O staining revealed reduced lipid droplet formation in lncRNA521141-silenced adipocytes (Fig. 2D). On day 10, the key adipocyte differentiation marker genes (C/EBPα, C/EBPβ, and FABP4) were significantly downregulated at both the mRNA (Fig. 2E) and protein levels (Fig. 2F and G). These results indicated that lncRNA521141 is required for adipocyte differentiation.

### lncRNA521141 regulates adipocyte differentiation by modulating PDGFRA expression

**Figure 3:**
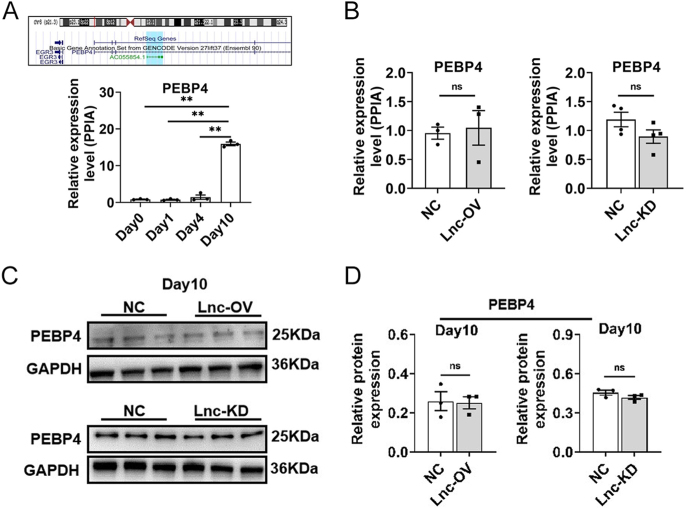
Impact of changes in lncRNA521141 expression on PEBP4. (A) The genomic location of lncRNA521141 relative to its host gene, PEBP4, is shown. The expression levels of PEBP4 in human preadipocytes were assessed by qPCR on days 0, 1, 4, and 10 of the differentiation program (three biologically independent samples per time point). (B) qPCR was used to evaluate PEBP4 mRNA expression following lncRNA521141 overexpression or knockdown PPIA mRNA levels were used as an internal control for normalization. (C and D) Western blot analysis was used to detect PEBP4 protein expression levels following lncRNA521141 overexpression or knockdown. Representative western blot images are shown for each group (three biological replicates per group). GAPDH protein levels were used as an internal control for normalization. The values are presented as mean ± SEM. **P < 0.01. A full color version of this figure is available at https://doi.org/10.1530/JOE-25-0360.

**Figure 4:**
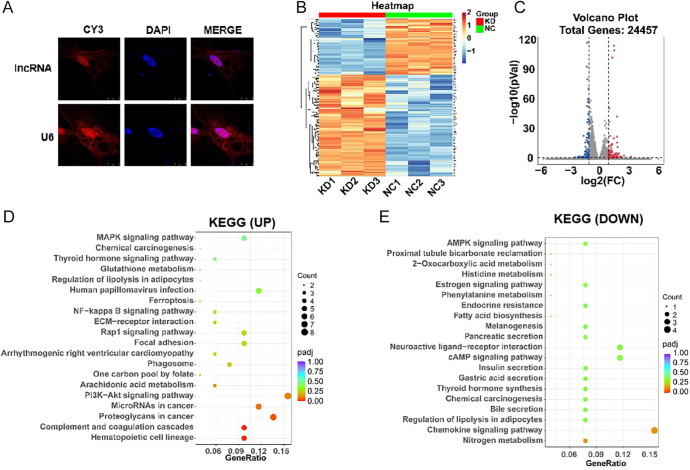
Potential regulatory pathways following lncRNA521141 knockdown in human adipocytes. (A) The spatial distribution of lncRNA521141 in mature adipocytes was visualized using FISH. (B) Heat map and cluster analyses were performed to illustrate the differentially expressed genes (DEGs) between the lnc-KD (KD1, KD2, and KD3) and NC (NC1, NC2, and NC3) groups in human mature adipocytes (*n* = 3 biological replicates per group). (C) A volcano plot was generated to depict the DEGs between lncRNA521141 knockdown and NC groups in human mature adipocytes. (D and E) Pathway analysis was conducted to elucidate the biological pathways associated with the upregulated or downregulated DEGs following lncRNA521141 knockdown in human adipocytes. A full color version of this figure is available at https://doi.org/10.1530/JOE-25-0360.

Several studies have reported that antisense lncRNAs can affect regulatory function by modulating the expression of their neighboring protein-encoding genes (20). Therefore, the association between lncRNA521141 and its neighboring gene PEBP4 was examined. Although qRT-PCR analysis revealed that PEBP4 mRNA expression gradually increased during human preadipocyte differentiation (Fig. 3A), overexpression or knockdown of lncRNA521141 did not significantly alter PEBP4 mRNA levels (Fig. 3B) or protein levels (Fig. 3C and D). To further elucidate the cellular localization of lncRNA521141, FISH was performed in mature adipocytes and the results showed that lncRNA521141 was predominantly expressed in the nuclei of mature adipocytes, with a small amount also detected in the cytoplasm (Fig. 4A).

**Figure 5:**
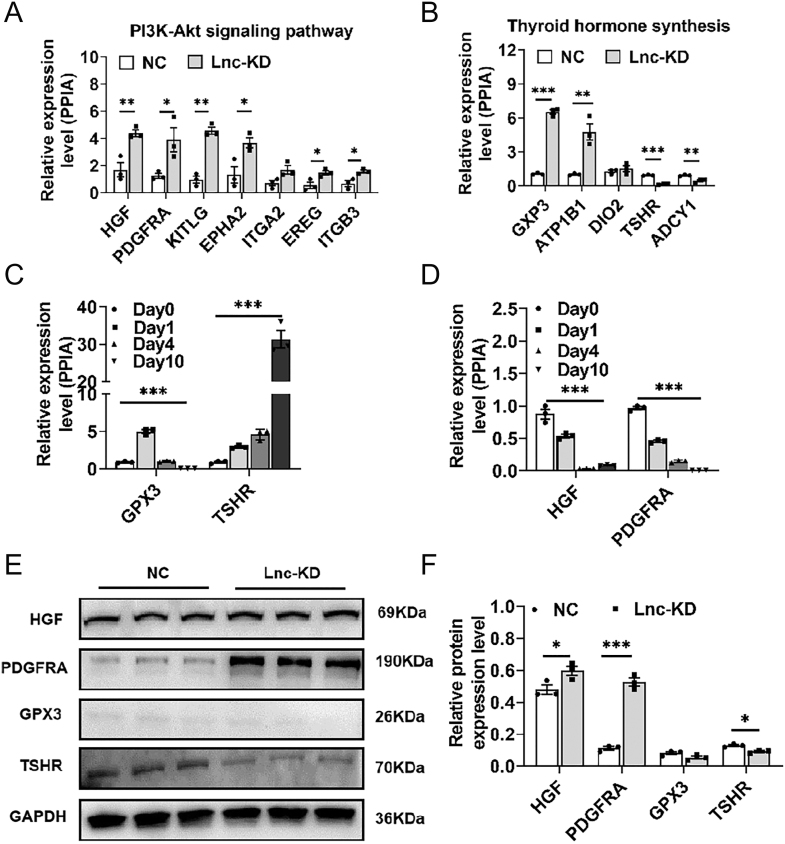
Validation of the DEGs involved in the potential regulatory pathways following lncRNA521141 knockdown in human adipocytes. (A and B) qPCR was employed to verify the DEGs implicated in the PI3K/Akt and thyroid hormone synthesis pathways (*n* = 3 biological replicates per group). (C and D) The mRNA expression levels of HGF, PDGFRA, GPX3, and TSHR were assessed via qPCR on days 0, 1, 4, and 10 of the differentiation program (*n* = 3 biological replicates per time point). (E) The protein expression levels of HGF, PDGFRA, GPX3, and TSHR were assessed by western blot analysis in human mature adipocytes and compared between the lncRNA521141 knockdown (lnc-KD) and NC groups on day 10 (*n* = 3 biological replicates per group). (F) Grayscale analysis of protein bands for HGF, PDGFRA, GPX3, and TSHR was performed to quantify the differences between the lncRNA521141 knockdown (lnc-KD) and NC groups. GAPDH protein levels were used as an internal control for normalization. The values are presented as mean ± SEM. *P < 0.05, **P < 0.01, ***P < 0.001.

**Figure 6:**
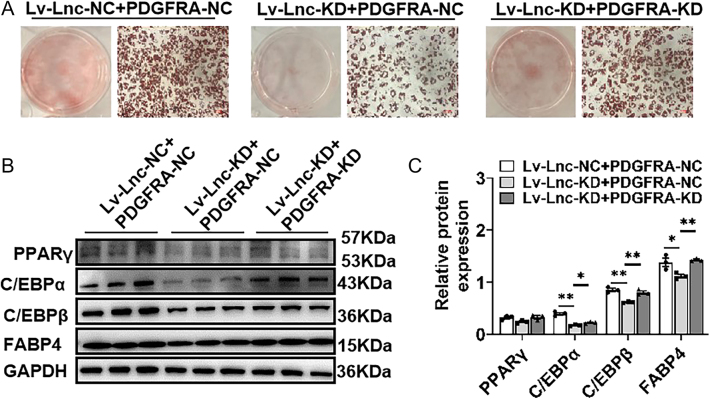
lncRNA521141 may regulate the adipogenic program via regulating PDGFRA expression. To validate whether PDGFRA is involved in the adipogenic program of white adipocytes mediated by lncRNA521141, PDGFRA was knocked down (PDGFRA-KD) in lncRNA521141-deficient cells (lnc-KD). (A) Oil Red O staining was performed to evaluate lipid morphology and droplet size after knockdown of PDGFRA in lnc-KD cells. (B and C) The protein expression levels of adipocyte differentiation marker proteins, including PPARγ, C/EBPα, C/EBPβ, and FABP4, were detected by western blot analysis. The representative western blots are shown. GAPDH served as an internal control for normalization at the protein level. The values are presented as mean ± SEM. *P < 0.05 and **P < 0.01. A full color version of this figure is available at https://doi.org/10.1530/JOE-25-0360.

To identify potential downstream pathways associated with lncRNA521141, the transcriptomes of the lncRNA521141 knockdown (lnc-KD) and NC groups on day 10 of differentiation were analyzed (Fig. 4B). Transcriptome sequencing revealed 206 DEGs upon lncRNA521141 silencing (log2 |fold change| ≥ 1, adj. *P* < 0.05), with 133 upregulated and 73 downregulated genes (Fig. 4C). Pathway analysis indicated that these DEGs were mainly enriched in pathways related to thyroid hormone synthesis, the PI3K/Akt signaling pathway, hematopoietic cell lineage, and chemokine signaling (Fig. 4D and E). The DEGs involved in the PI3K/Akt signaling pathway included hepatocyte growth factor (HGF), PDGFRA, Kit ligand (KITLG), erythropoietin-producing hepatocellular A 2 (EPHA2), integrin subunit alpha 2 (ITGA2), epiregulin (EREG), and integrin subunit beta 3 (ITGB3), while those in the thyroid hormone synthesis pathway included glutathione peroxidase 3 (GPX3), ATPase Na+/K+ transporting subunit beta 1 (ATP1B1), iodothyronine deiodinase 2 (DIO2), thyroid-stimulating hormone receptor (TSHR), and adenylate cyclase 1 (ADCY1). qRT-PCR confirmed that the expression changes of DEGs at the mRNA level were consistent with the sequencing results (Fig. 5A and B). Previous studies have reported that HGF (21), PDGFRA (22, 23, 24), GPX3 (25, 26, 27), and TSHR are involved in developmental adipose lineage, adipocyte differentiation, and adult WAT homeostasis. Moreover, qRT-PCR analysis revealed that the expression levels of HGF and PDGFRA were downregulated during adipocyte differentiation program (Fig. 5D). Western blot analysis also verified an upregulated expression trend of HGF and PDGFRA following lncRNA521141 knockdown, with PDGFRA exhibiting a more pronounced trend (an approximately fivefold increase) (Fig. 5E and F). These results support the notion that PGDFRA is a potential target of lncRNA521141. To further investigate the role of PDGFRA, the expression level of PDGFRA was knocked down in lncRNA521141-deficient cells. Oil Red O staining revealed that knockdown of PDGFRA alleviated the inhibition of adipocyte differentiation caused by lncRNA521141 silencing (Fig. 6A). In addition, PDGFRA silencing increased the protein expression levels of adipocyte differentiation marker genes (C/EBPα, C/EBPβ, and FABP4) (Fig. 6B and C). Collectively, these findings suggested that lncRNA521141 may regulate adipogenic differentiation through modulating PDGFRA expression.

## Discussion

Visceral obesity poses a significant burden on societal welfare, thereby fueling interest in understanding the mechanisms of adipocyte differentiation. This understanding is crucial for reducing the prevalence of obesity and mitigating its detrimental physiological effects. In recent years, human preadipocytes have served as a useful *in vitro* model for exploring the molecular target in adipocyte differentiation and potential treatments for obesity (28, 29). There have been an increasing number of studies on the functional role of lncRNAs, such as lncAATBC (30), lnc-ADAIN (31), lnc-GALNTL6-4 (32), and SNHG12 (33), in human adipocytes and WAT metabolism. Thus, targeting the adipogenesis- or obesity-related lncRNAs may provide potential strategies for the prevention and improvement of obesity and related metabolic disorders.

The present study characterized the expression profiles of lncRNAs throughout human adipocyte differentiation, utilizing both visceral preadipocytes and fully differentiated mature adipocytes as shown in Supplementary Fig. S1. Compared with preadipocytes, lncRNA521141 was significantly upregulated in human mature adipocytes. To date, the expression pattern and role of lncRNA521141 in adipocyte differentiation or WAT homeostasis have remained unexplored. The present study revealed a progressive upregulation of lncRNA521141 during the differentiation process (Fig. 1A), suggesting its potential involvement in the regulation of human preadipocyte differentiation. lncRNA521141 overexpression did not significantly affect adipocyte differentiation (Fig. 1B, C, D, E, F, G, H), but knockdown of lncRNA521141 demonstrated that lncRNA521141 is a required regulator of human visceral preadipocyte differentiation (Fig. 2). Similar to lncRNA521141, other lncRNAs, such as lnc-leptin, are necessary but not sufficient to promote adipogenesis (34). Although lnc-leptin expression gradually increases with the progression of adipocyte differentiation, its overexpression fails to enhance the expression of leptin or other adipocyte markers. Conversely, knockdown of lnc-leptin significantly blocks adipogenesis.

The important mechanism of antisense lncRNA is to regulate the expression of neighboring genes, mainly through chromatin modification (35), mRNA splicing (36), and mRNA translation (37, 38). Because antisense lncRNAs may regulate neighboring genes (39), the present study investigated whether the neighboring gene PEBP4 is a direct target of lncRNA521141. Overexpression or silencing of lncRNA521141 had no significant effect on the mRNA and protein levels of PEBP4 as shown in Figs 3B and 4D. Transcriptome sequencing and pathway analysis revealed that the thyroid hormone synthesis pathway and the PI3K/Akt signaling pathway are closely related to the inhibition of adipocyte differentiation upon lncRNA521141 knockdown (Fig. 4B, C, D, E). On the basis of literature reviews and validation analysis, our present study focused on HGF, PDGFRA, GPX3, and TSHR. Compared to other potential targets, the mRNA and protein expression changes of PDGFRA after lncRNA521141 knockdown were the most significant, making PDGFRA the primary focus. PDGFRA, a membrane-bound tyrosine kinase receptor expressed in perivascular stromal cells across various tissues, is commonly used as a cell surface marker for identifying mouse and human adipose progenitors (5, 40, 41). Recent studies have reported that PDGFRA acts as a cell-autonomous inhibitor of adipocyte differentiation and plays a critical role in the transition from adipose progenitors to mature adipocytes (23). In addition to its role in adipocyte lineage commitment, PDGFRA decreases during the differentiation program of preadipocytes or cell lines (42) and exerts an inhibitory effect on lipid accumulation and adipogenic gene expression (43, 44). The rescue experiment revealed that PDGFRA knockdown in lncRNA521141-deficient cells alleviated the inhibitory effect on adipocyte differentiation caused by lncRNA521141 silencing and upregulated the protein expression levels of adipogenic markers (Fig. 6). Considering the previously reported browning effect of PDGFRA (44), it cannot be excluded that the inhibitory effect of lncRNA521141 on adipocyte differentiation may also partly lead to a browning phenotype.

The subcellular localization of lncRNAs is a determinant of their diverse mechanisms of action (45). FISH analysis confirmed that lncRNA521141 was localized predominantly in the nuclei of differentiated adipocytes, suggesting its role in adipogenesis at the transcriptional level (Fig. 4A). Other examples of adipogenesis regulation include nuclear-expressed lncRNA Plnc1, which increases PPARγ2 transcriptional activity by reducing CpG methylation in the PPARγ2 promoter (46). Similarly, MIR31HG inhibition decreases the enrichment of H3K4me3 and acetylation in the promoter of FABP4, thereby suppressing FABP4 expression and adipocyte differentiation (47). These findings provide insights into the potential molecular mechanisms of lncRNA521141 in adipogenesis. However, the proteins that bind to lncRNA521141 to regulate downstream pathways remain unknown. Given the enrichment of H3K27ac in PDGFRA, as revealed by whole-genome H3K27ac profiling (48), future studies should focus on elucidating the specific mechanisms by which lncRNA521141 regulates PDGFRA expression, thereby providing a deeper understanding of its role in adipogenesis.

In summary, the present study highlights the role of lncRNA521141 in human adipocyte differentiation. Functionally, knockdown of lncRNA521141 revealed that lncRNA521141 is required for adipocyte differentiation, which was significantly impaired upon its depletion. Mechanistically, the inhibitory effect of lncRNA521141 knockdown on adipocyte differentiation was mediated through regulation of PDGFRA expression. The present study identified a novel lncRNA-mediated regulatory network in the modulation of human adipocyte differentiation. However, the present study had several limitations. Even though the *in vitro* experiments using human preadipocytes clearly demonstrated these effects, the physiological relevance of lncRNA521141 *in vivo* remains uncertain, owing to the lack of data from human adipose tissue samples or *in vivo* animal models. The regulatory network observed *in vitro* may not be fully validated in visceral WAT. Although the present findings have improved the understanding of adipogenesis and provided potential targets for obesity and related metabolic diseases, the clinical value of lncRNA521141 as a therapeutic target remains to be validated in the future.

## Supplementary materials









## Declaration of interest

The authors declare that there is no conflict of interest that could be perceived as prejudicing the impartiality of the work reported.

## Funding

This study was supported by the National Natural Science Foundation of China (81800772 and 82170823), Jiangsu Province Natural Science Foundation (Grant Nos. BK20180146 and BK20191126), Jiangsu Association for Science & Technology Youth Science & Technology Talents Lifting Project (TJ-2022-005), and Nanjing Medical Science and Technique Development Foundation (YKK21166).

## Author contribution statement

Lingxia Pang, Hui Liang, and Lijian Xu conceived the study. Qiong Wu and Lianghui You carried out the experiments and were in charge of data analysis. Qiong Wu and Lianghui You drafted the manuscript. Shibo Lin, Tingting Yu, Rui Yin, and Xinrong Ye helped with data interpretation. Lingxia Pang and Lianghui You revised the manuscript and approved the submission. Financial support was provided by Lingxia Pang and Lianghui You. All authors approved the final version of the paper.
