# Supplementary material for: The impact and potential mechanisms of long noncoding RNA ENST00000521141.1 on human white adipocyte differentiation
Source: J Endocrinol. 2026 Apr 21;269(1):e250360. doi: 10.1530/JOE-25-0360 (PMC13130826; doi:10.1530/JOE-25-0360)
Supplement: Supplementary file 1 [file supplementary_materials.pdf]

Supplemental materials and Results

1. Supplemental materials

1.1 LncRNA microarray and bioinformatics analysis

The extracted total RNA from adipocytes was sent to Shanghai Kangcheng Bioengineering Co., Ltd. for chip detection. The LncRNAs microarray experiment adopted the third-generation Arraystar Human LncRNA Microarray V3.0 chip technology provided by Kangcheng Company. After removing rRNA from the total RNA using the mRNA-ONLY Eukaryotic mRNA Isolation Kit (Epicentre), purified mRNA was obtained. Subsequently, random primer method was used for amplification, and the mRNA was transcribed into fluorescent cRNA of the full transcript length. The labeled cRNA was hybridized to the Human LncRNA Array v3.0 (8×60K, Arraystar). A chip scanner (Agilent Scanner G22505C) was used to scan the fluorescence intensity of the chip, and the experimental results were converted into microarray images by software (Agilent Feature Extraction software, version 11.0.1.1). The GeneSpring GX v12.0 software package (Agilent Technologies) was applied for data standardization and processing. The screening criteria for differentially expressed genes were as follows: Fold-Change ≥ 2 and P-value < 0.05. For the functional enrichment analysis of the obtained differentially expressed protein-coding genes, the Database for Annotation, Visualization and Integrated Discovery (DAVID, http://david.abcc.ncifcrf.gov/) was used. Gene Ontology (GO) enrichment (biological process, cellular component, molecular function) and KEGG pathway enrichment were evaluated by hypergeometric test; terms with P < 0.05 were considered significant.

1.2 Screening of candidate lncRNAs

First, lncRNAs were screened based on the following criteria: small intra-group fold change, large inter-group fold change (Fold-Change ≥ 5) between human mature adipocytes and preadipocytes, P-value < 0.05, and raw intensity ≥ 150. From these screened lncRNAs, 10 up-regulated and 10 down-regulated ones were selected for verification. Subsequently, the sequences of these lncRNAs were retrieved from

databases such as Ensembl, and primers were designed using the online software Primer Premier 3.

1.3 qRT-PCR primer design and synthesis

The sequences of lncRNAs were retrieved from databases such as Ensembl, NCBI, and UCSC. Primer sequences were designed using the Primer 3.0 online primer design website (http://sourceforge.net/projects/primer3/), and then aligned with the NCBI Basic Local Alignment Search Tool (BLAST) to ensure the uniqueness of the products. The primers were synthesized by Shanghai Generay Biotechnology Co., Ltd. The primer sequences are shown in **Table S1**.

Databases for lncRNA sequence retrieval:

- http://www.ensembl.org/index.html
- https://www.ncbi.nlm.nih.gov/
- http://genome.ucsc.edu/

Software for sequence homology comparison:

- http://www.ncbi.nlm.nih.gov/cgi-bin/BLAST

Database for lncRNA bioinformatics analysis:

- http://genome.ucsc.edu/index.html

2. Results

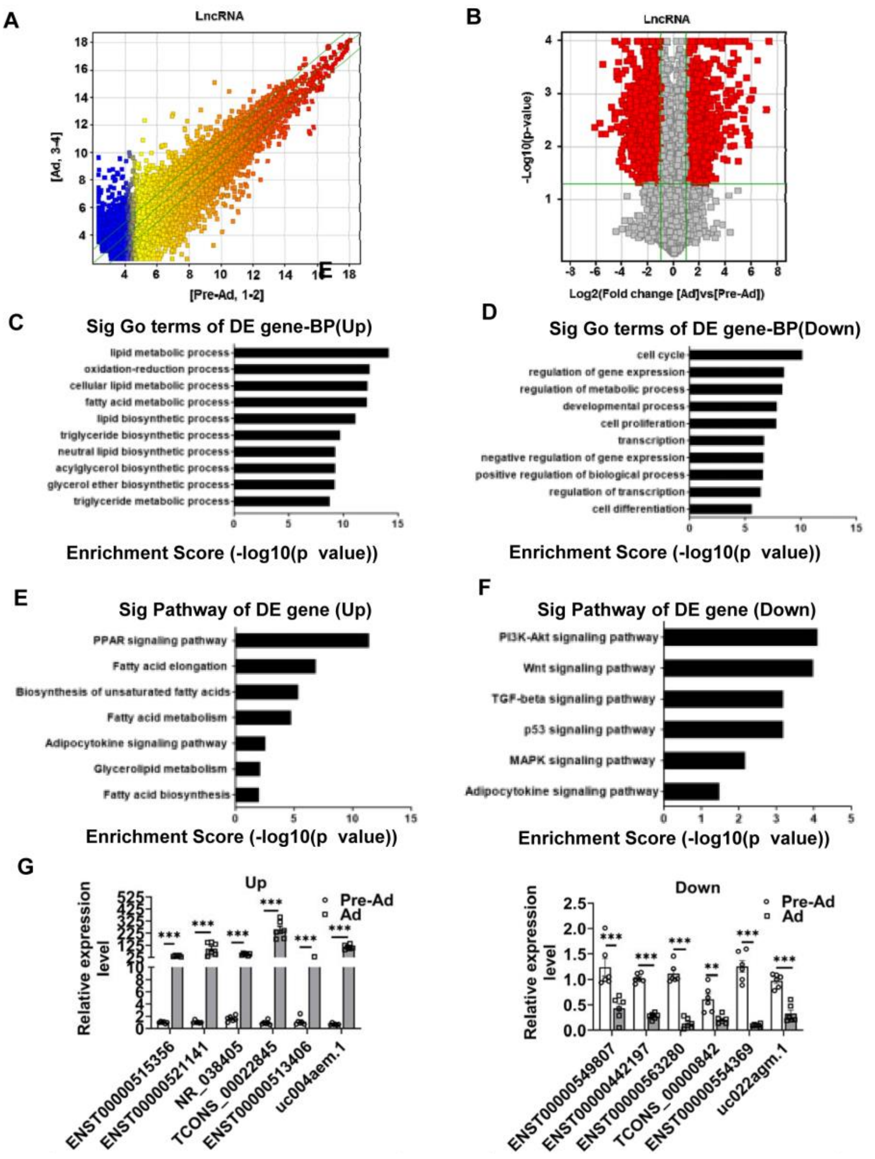

Figure S1. LncRNA-related Microarray Data. (A): Scatter plots of differentially expressed lncRNAs by microarray. (B): Volcano plots of differentially expressed lncRNAs by microarray. (C-D): GO analysis of differentially expressed mRNAs. (E-F): Pathway analysis of differentially expressed mRNAs. (G): Verification of screened candidate lncRNAs in human preadipocytes and mature adipocytes by qRT-PCR (n=6). Values are presented as the mean ± SEM. **, P<0.01;***, P<0.001.

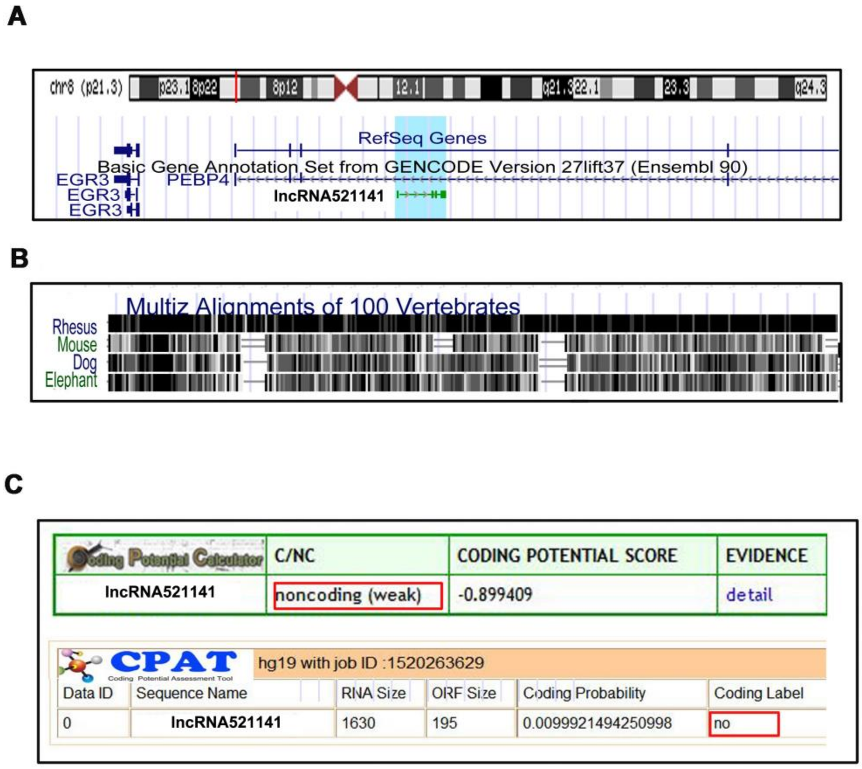

Figure S2. Basic characteristics of lncRNA521141. (A): Genomic location of lncRNA521141 and its neighboring gene PEBP4 from UCSC genome browser. (B): conservative analysis of partial sequence of lncRNA521141 among different species. (C): Prediction of the coding potential of lncRNA521141 using the Coding Potential Calculator (CPC) and Coding Potential Assessment Tool (CPAT).

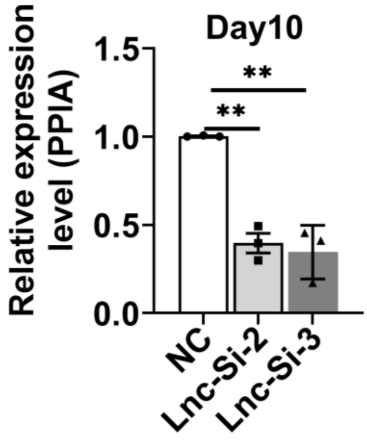

Figure S3. The inhibited efficiency of the small interference RNA sequence (Si-2 and Si-3) on lncRNA521141. Data are mean ± SEM of three biological independent samples. **, P<0.01.
